# Elimination of Left-Right Reciprocal Coupling in the Adult Lamprey Spinal Cord Abolishes the Generation of Locomotor Activity

**DOI:** 10.3389/fncir.2017.00089

**Published:** 2017-11-24

**Authors:** J. A. Messina, Alison St. Paul, Sarah Hargis, Wengora E. Thompson, Andrew D. McClellan

**Affiliations:** ^1^Division of Biological Sciences, University of Missouri, Columbia, MO, United States; ^2^Interdisciplinary Neuroscience Program, University of Missouri, Columbia, MO, United States

**Keywords:** locomotion, central pattern generators, oscillators, coordination, coupling

## Abstract

The contribution of left-right reciprocal coupling between spinal locomotor networks to the generation of locomotor activity was tested in adult lampreys. Muscle recordings were made from normal animals as well as from experimental animals with rostral midline (ML) spinal lesions (~13%→35% body length, BL), before and after spinal transections (T) at 35% BL. Importantly, in the present study actual locomotor movements and muscle burst activity, as well as other motor activity, were initiated in whole animals by descending brain-spinal pathways in response to sensory stimulation of the anterior head. For experimental animals with ML spinal lesions, sensory stimulation could elicit well-coordinated locomotor muscle burst activity, but with some significant differences in the parameters of locomotor activity compared to those for normal animals. Computer models representing normal animals or experimental animals with ML spinal lesions could mimic many of the differences in locomotor activity. For experimental animals with ML and T spinal lesions, right and left rostral hemi-spinal cords, disconnected from intact caudal cord, usually produced tonic or unpatterned muscle activity. Hemi-spinal cords sometimes generated spontaneous or sensory-evoked relatively high frequency “burstlet” activity that probably is analogous to the previously described *in vitro* “fast rhythm”, which is thought to represent lamprey locomotor activity. However, “burstlet” activity in the present study had parameters and features that were very different than those for lamprey locomotor activity: average frequencies were ~25 Hz, but individual frequencies could be >50 Hz; burst proportions (BPs) often varied with cycled time; “burstlet” activity usually was not accompanied by a rostrocaudal phase lag; and following ML spinal lesions alone, “burstlet” activity could occur in the presence or absence of swimming burst activity, suggesting the two were generated by different mechanisms. In summary, for adult lampreys, left and right hemi-spinal cords did not generate rhythmic locomotor activity in response to descending inputs from the brain, suggesting that left-right reciprocal coupling of spinal locomotor networks contributes to both phase control and rhythmogenesis. In addition, the present study indicates that extreme caution should be exercised when testing the operation of spinal locomotor networks using artificial activation of isolated or reduced nervous system preparations.

## Introduction

Within the central nervous system (CNS) of many animals, central pattern generators (CPGs) are able to produce the basic motor patterns for rhythmic behaviors in the absence of sensory feedback, although sensory inputs are necessary to modulate motor patterns to adapt them to the ongoing needs of the animal (reviewed in Orlovsky et al., 1999). The CPGs often consist of several “local control centers” or “CPG modules” that are distributed in the CNS and that each controls the rhythmic movements of different structures of the body. A coordinating system couples the different CPG modules to regulate the relative timing of the overall rhythmic motor patterns (reviewed in Skinner and Mulloney, 1998; Hill et al., 2003). Alternating motor patterns, such as left-right alternation or flexor-extensor alternation, are thought to be generated by “half-center” networks in which two CPG modules are connected by reciprocal inhibition (Friesen, 1994).

**Figure 1:**
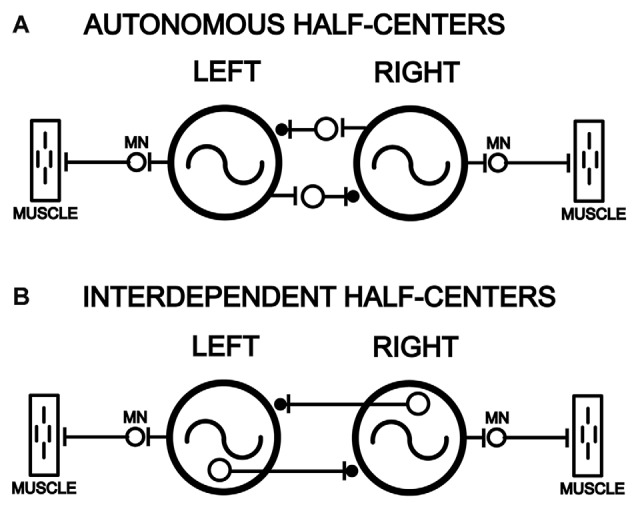
Models of left-right pairs of spinal cord oscillators that are coupled by relatively strong reciprocal inhibition in parallel with weaker reciprocal excitation (–|●), as is the case for the lamprey spinal locomotor central pattern generators (CPGs; Hagevik and McClellan, 1994; MN, motoneuron pool). **(A)** Left and right spinal oscillators that are autonomous and not dependent on left-right reciprocal connections for rhythmogenesis and generation of rhythmic locomotor burst activity. **(B)** Left and right spinal oscillators that are interdependent and require left-right coupling for rhythmogenesis and the generation of locomotor burst activity.

For some animals, the CPG modules are rhythmogenic or autonomous (Figure 1A) when isolated from other CPG modules, while in other animals CPG modules are interdependent (Figure 1B) and need to be connected to other modules to function properly. For rhythmic invertebrate behaviors, the situation appears to be mixed. For some invertebrates (crayfish and *Clione*), CPG modules can function autonomously when isolated from other modules (Murchison et al., 1993; Arshavsky et al., 1998). However, for other animals (leech), isolated CPG modules are unable to generate spontaneous or pharmacologically-induced rhythmic motor activity (Friesen and Hocker, 2001).

Mammalian quadrupedal locomotion is thought to be generated by spinal “local control centers” or CPG modules that each control the rhythmic movements of an individual limb (reviewed in Orlovsky et al., 1999). First, application of strychnine to the isolated neonatal rat spinal cord converts pharmacologically-induced left-right alternating locomotor-like burst activity to synchronous bursting (Cowley and Schmidt, 1995; also see Jovanović et al., 1999; for similar results in mudpuppy). These results suggest that right and left CPG modules are rhythmogenic. For the isolated neonatal mouse spinal cord, spontaneous or induced (electrically or pharmacologically) rhythmic flexor or extensor bursts can occur without antagonistic motor activity (Whelan et al., 2000; also see Cheng et al., 1998; for similar results in mudpuppy), suggesting that flexor and extensor CPGs are rhythmogenic. However, there are alternative interpretations for these types of results (see “Discussion” section).

Second, for the cat, right or left lumbar hemi-spinal cord regions, surgically separated by a midline spinal lesion, can produce locomotor-like movements and muscle burst activity for the corresponding hindlimb (Kato, 1990). Surgically isolated right or left lumbar spinal networks from neonatal mice or rats can generate rhythmic locomotor-like burst activity in response to bath-applied pharmacological agents (Kudo and Yamada, 1987; Tao and Droge, 1992; Bracci et al., 1996; Cowley and Schmidt, 1997; Kjaerulff and Kiehn, 1997; Kremer and Lev-Tov, 1997; Bonnot and Morin, 1998; Whelan et al., 2000; Nakayama et al., 2002; also see Cheng et al., 1998). In contrast, results from experiments in which *in vitro* neonatal rat spinal cord was activated by descending brain-spinal cord pathways, instead of pharmacological activation, suggest that commissural connections in the thoracolumbar spinal cord are critical for generation of locomotor-like burst activity (Cowley et al., 2009).

The *in vitro* embryonic chick spinal cord can generate episodes of locomotor-like activity (O’Donovan, 1989). Following mid-sagittal lesions in the lumbosacral spinal cord, left or right spinal CPG modules still generate rhythmic burst activity (Ho and O’Donovan, 1993), similar to the results described above for mammals.

For the spinal turtle, unilateral sensory stimulation of different areas of the lower body elicits different variations of the rhythmic scratch reflex for the ipsilateral hindlimb (reviewed in Stein et al., 1998b), suggesting that separate left and right spinal CPG modules control scratching responses for each hindlimb. However, several additional results suggest that contralateral spinal circuitry contributes to ipsilateral scratch motor pattern generation (Stein et al., 1995, 1998a; reviewed in Stein et al., 1998b).

Most fish and some amphibians locomote (swim) by undulatory movements that are produced by muscle burst activity with two components (reviewed in Grillner and Kashin, 1976): (1) left-right alternating burst activity that produces left-right bending of the body at each segmental level; and (2) a rostrocaudal phase lag for ipsilateral burst activity that causes body undulations (S-waves) to propagate toward the tail and generate forward thrust. The swim motor pattern is generated by spinal CPG modules that are distributed bilaterally and longitudinally in the spinal cord and coupled by a coordinating system (reviewed in McClellan, 1996).

For lamprey swimming, the mechanisms for left-right alternation and rostrocaudal phase lags have been studied extensively. First, left-right alternating locomotor activity is produced by left and right spinal CPG modules that are coupled by relatively strong reciprocal inhibition in parallel with weaker reciprocal excitation (see Figure 10A; Cohen and Harris-Warrick, 1984; Alford and Williams, 1989; Hagevik and McClellan, 1994; also see Roberts et al., 1985; for similar conclusions for *Xenopus*). At least part of the reciprocal inhibition appears to be mediated by crossed-contralaterally projecting interneurons (CCI’s), a class of spinal commissural interneurons whose axons primarily project contralaterally and caudally for ~2–10 segments (reviewed in Buchanan, 2001). Evidence for excitatory commissural interneurons also has been found (Biró et al., 2008). Second, physiological and computer modeling studies suggest that rostrocaudal phase lags are mediated mainly by relatively short-distance asymmetrical excitatory coupling between ipsilateral CPG modules that is stronger in the descending direction than the ascending direction (see Figure 10A; Hagevik and McClellan, 1994, 1999; McClellan and Hagevik, 1999; reviewed in McClellan, 1996). Reciprocal inhibition mediated by the CCI’s, which are glycinergic, probably does not contribute substantially to rostrocaudal phase lags because in the presence of strychnine, phase lags are present and relatively close to normal values (Hagevik and McClellan, 1994). Longer distance ipsilateral coupling is present in the lamprey spinal cord but becomes progressively weaker with increasing distance (McClellan and Hagevik, 1999).

**Figure 2:**
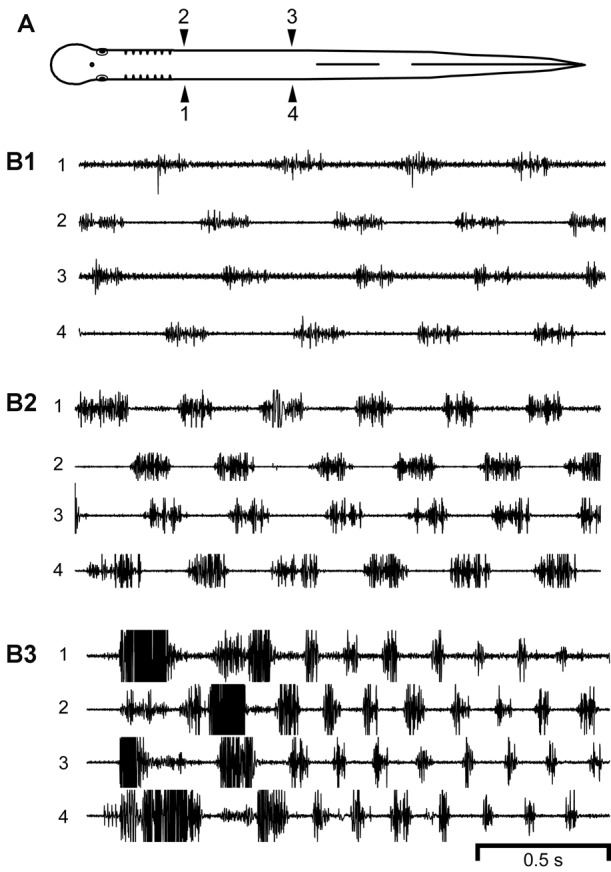
**(A)** Diagram of a normal adult lamprey showing bilateral muscle recording electrodes at 25% body length (BL, normalized distance from anterior tip of the head; 1, 2) and 45% BL (3, 4). **(B)** Locomotor muscle burst activity with different cycle times (CTs). During relatively **(B1)** slow (CT ≈ 460 ms, Freq ≈ 2.17 Hz), **(B2)** medium (CT ≈ 330 ms, Freq ≈ 3.03 Hz), or **(B3)** fast (CT ≈ 164 ms, Freq ≈ 6.10 Hz) swimming, locomotor muscle burst activity was characterized by left-right alternation (1↔2, 3↔4) and a rostrocaudal phase lag (1→4, 2→3). Note the decrease in CT for faster swimming. Some of the muscle action potentials were clipped in **(B2,B3)**.

**Figure 3:**
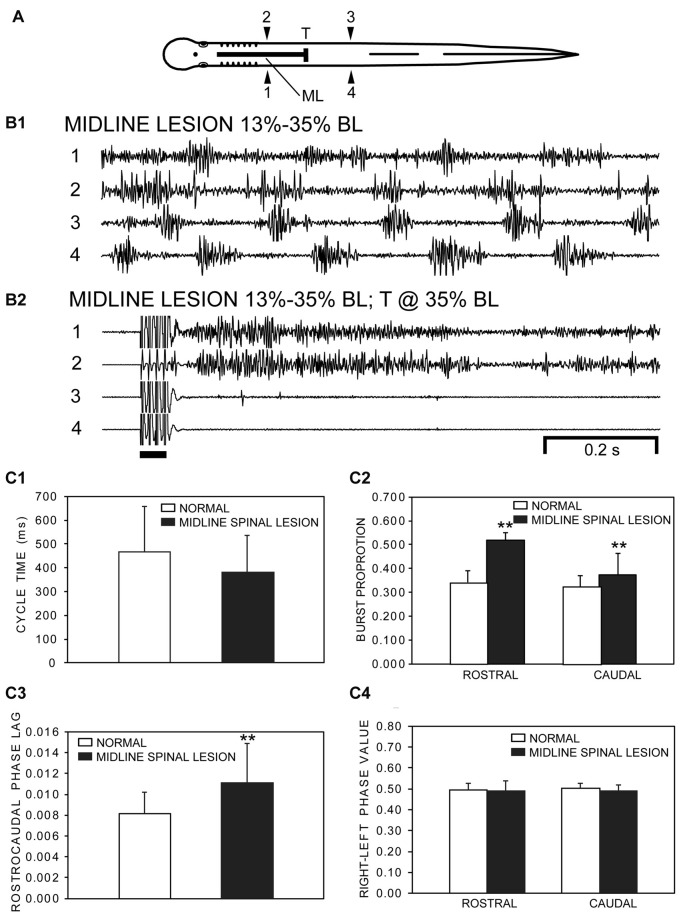
**(A)** Diagram of an experimental adult lamprey showing muscle recording electrodes at 25% BL (1, 2) and 45% BL (3, 4), longitudinal midline (ML) spinal cord lesion from 13% to 35% BL (thick horizontal line), and spinal cord transection (T) at 35% BL. **(B)** Muscle activity. **(B1)** Following a ML spinal cord lesion alone, locomotor muscle burst activity during swimming (CT ≈ 211 ms, Freq ≈ 4.74 Hz) was characterized by left-right alternation of muscle activity in the rostral (1↔2) and caudal (3↔4) body as well as a rostrocaudal phase lag for ipsilateral activity (1→4, 2→3). **(B2)** Subsequently, following a spinal transection at 35% BL for the same animal as in “**B1**”, stimulation of the oral hood (bar) elicited tonic muscle activity in the rostral body (1, 2), while movements and muscle activity were absent in the caudal body (3, 4). **(C)** Parameters of locomotor muscle burst activity (bars = means; vertical lines = SDs) during swimming for normal animals (open bars; *n* = 15) and for experimental animals with rostral longitudinal ML spinal cord lesions alone (filled bars; *n* = 21; see “Materials and Methods” section): **(C1)** CTs; **(C2)** burst proportions (BPs) for rostral and caudal locomotor muscle burst activity; **(C3)** intersegmental rostrocaudal phase lags; and **(C4)** right-left phase values for rostral and caudal locomotor muscle burst activity. Statistics: **p < 0.01; unpaired *t*-tests with Welch correction, when necessary, or Kruskal-Wallis with Dunn’s multiple comparisons post-test).

**Figure 4:**
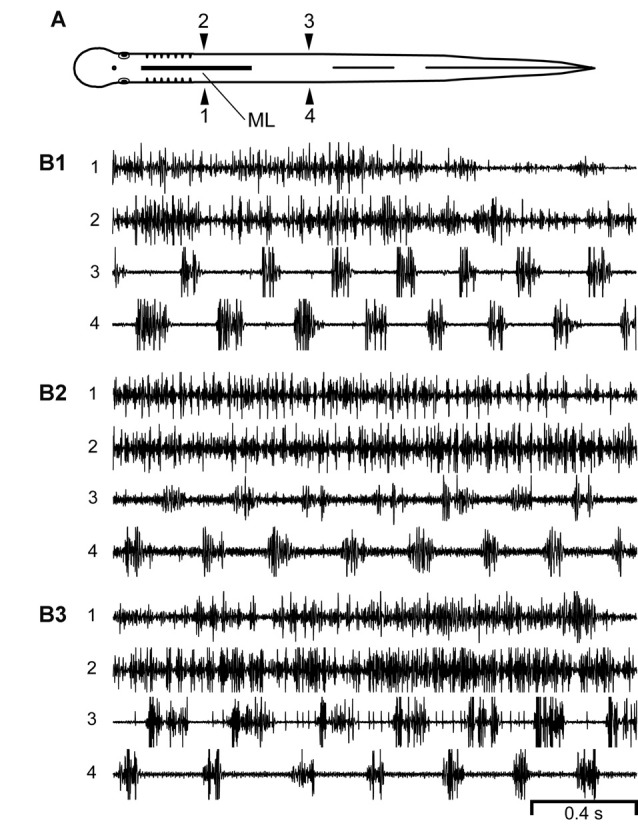
**(A)** Diagram of an experimental adult lamprey showing muscle recording electrodes at 25% BL (1, 2) and 45% BL (3, 4), and longitudinal midline (ML) spinal cord lesion from 13% to 35% BL (thick horizontal line). **(B)** Muscle activity during swimming for three different animals. For these particular animals (*n* = 10; see text) there was left-right alternation of caudal locomotor muscle burst activity (3↔4), while rostral activity usually was either tonic or consisted of relatively high-frequency “burstlet” activity (1 and 2; see text and Figures 5–7 for further descriptions of “burstlet” activity).

**Figure 5:**
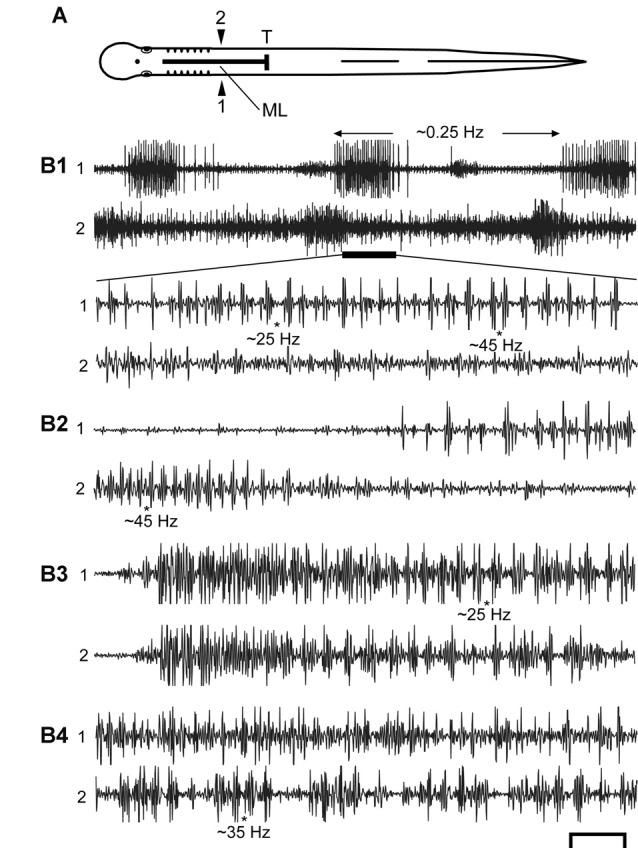
**(A)** Diagram of an experimental adult lamprey showing muscle recording electrodes at 25% BL (1, 2), longitudinal ML spinal cord lesion from 13 to 35% BL (thick horizontal line), and spinal cord transection (T) at 35% BL. Muscle recording electrodes at 45% BL are not shown for simplicity (see Figures 3A, 4A). **(B)** Examples of spontaneous or sensory-evoked muscle activity for different preparations following both spinal lesions. **(B1)** (upper) Very slow “burst” activity (~0.25 Hz, between horizontal arrows), and the activity during the horizontal black bar is expanded (lower) showing that the longer “bursts” consist of repetitive “bustlets” occurring at relatively high frequencies (see Figures 6A1–C1). **(B2–B4)** Examples of episodes of “burstlet” activity for other animals. Approximate instantaneous “burstlet” frequencies (*) shown below selected parts of the recordings. Scale bar: 1.0 s (**B1** upper), 100 ms (other recordings).

**Figure 6:**
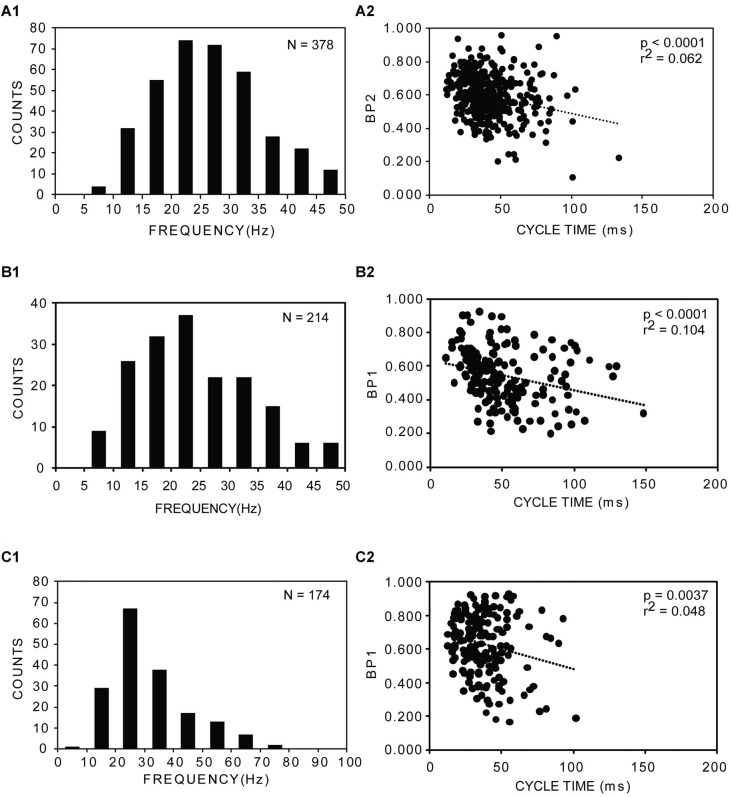
**(A1–C1)** Distributions of frequencies of rostral muscle “burstlet” activity (25% BL) for three experimental animals following both a longitudinal ML spinal cord lesion and spinal cord transection (**A** = left, rostral recording channel; **B,C** = right, rostral recording channel; see 1 and 2 in Figure 5A). Average frequencies: **(A1)** 28.4 ± 12.3 Hz (20 frequencies >50 Hz, not shown); **(B1)** 26.6 ± 12.8 Hz (5 frequencies >50 Hz, not shown); and **(C1)** 31.5 ± 13.9 Hz. *N* = total number of analyzed cycles for each animal. **(A2–C2)** Plots of BP vs. CT for the corresponding animals shown in **(A1–C1)** (BP1 = BP for channel 1; BP2 = BP for channel 2; see 1 and 2 in Figure 5A). Dotted lines, *p* values and *r*^2^ values indicate results from regression analysis.

**Figure 7:**
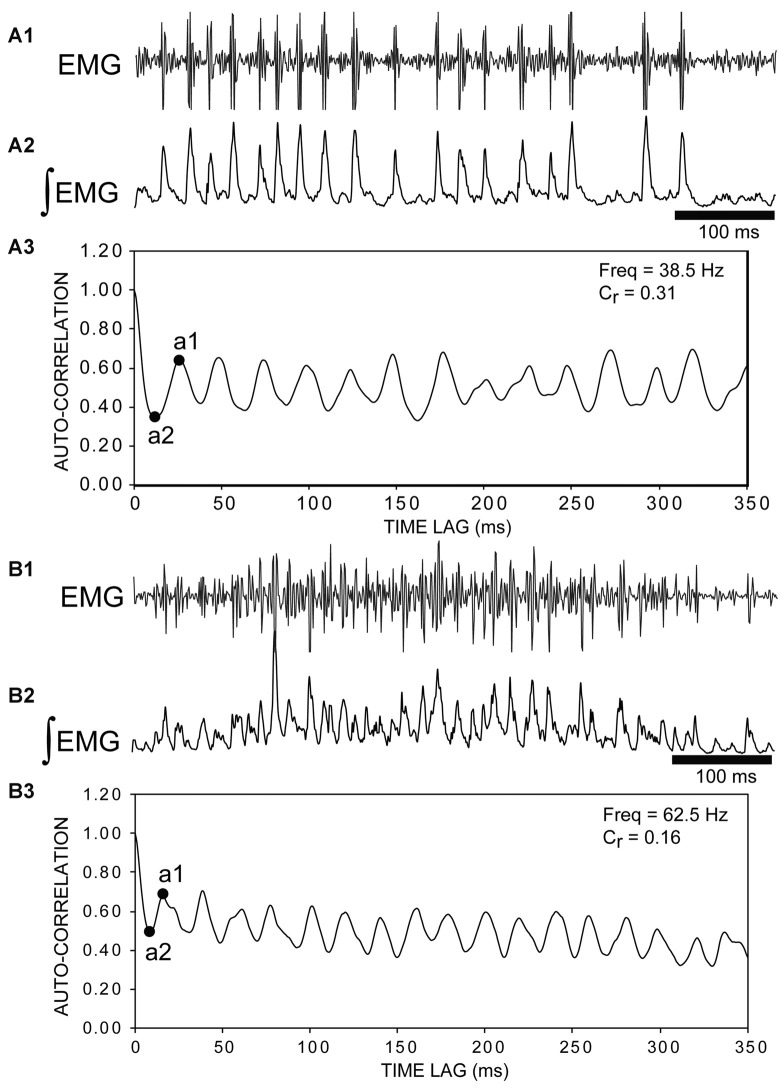
**(A1)** Raw muscle “burstlet” activity and **(A2)** integrated “burstlet” activity (τ = 3 ms) recorded from the right, rostral body (25% BL) following a rostral ML spinal lesion and spinal transection (T) (e.g., see channel 2 in Figure 5A). **(A3)** Autocorrelation of activity in “**A2**”. The coefficient of rhythmicity (*C*_r_ = 0.31) was calculated using the amplitude of the first trough (a2) and the amplitude of the second peak (a1) (see “Materials and Methods” section). The “burstlet” frequency was calculated from the inverse of the x-axis coordinate (time lag) for point “a1” and was equal to ~38.5 Hz. **(B1)** Raw muscle “burstlet” activity and **(B2)** integrated “burstlet” activity (τ = 3 ms) recorded from the right, rostral body (25% BL) from a different animal than in “**A**” following a ML spinal lesion and spinal transection. **(B3)** Autocorrelation of activity in “**B2**”. The coefficient of rhythmicity was 0.16. The frequency calculated from point “a1” was 62.5 Hz, but the mid-point of the second peak (~20 ms) corresponded to a frequency of ~50 Hz.

**Figure 8:**
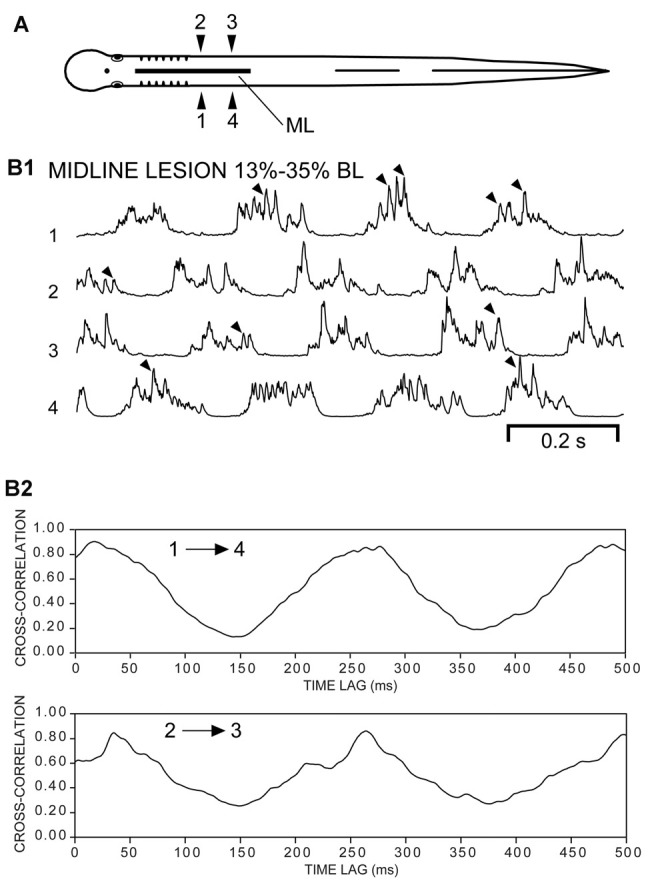
**(A)** Diagram of an experimental adult lamprey showing muscle recording electrodes at 25% BL (1, 2) and 30% BL (3, 4), and longitudinal ML spinal cord lesion from 13% to 35% BL (thick horizontal line). **(B1)** Integrated muscle burst activity (τ = 5 ms) characterized by left-right alternation (1↔2, 3↔4) and a rostrocaudal phase lag (1→4, 2→3). Note the “burstlet” activity (arrowheads) superimposed on the longer locomotor bursts. **(B2)** Cross-correlation of activity in **(B1)** indicating a phase delay of ~17 ms (1→4, upper plot, first peak) and a CT ≈ 250 ms (Freq ≈ 4.0 Hz), which corresponded to an intersegmental rostrocaudal phase lag of ~0.011.

**Figure 9:**
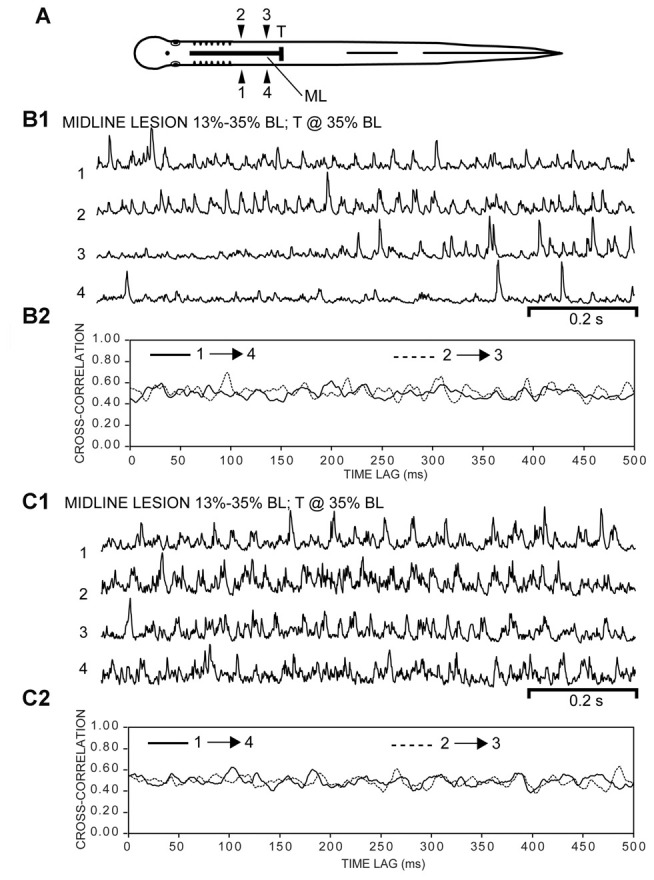
**(A)** Diagram of experimental adult lamprey (same animal as in Figure 8) showing muscle recording electrodes at 25% BL (1, 2) and 30% BL (3, 4), longitudinal ML spinal cord lesion from 13 to 35% BL (thick horizontal line), and spinal transection (T) at 35% BL. **(B1)** Integrated “burstlet” activity (τ = 3 ms) and **(B2)** corresponding cross-correlation plot of this activity for recording channels 1→4 and 2→3. For the initial part of the recording, note the “burstlet” activity for one ipsilateral channel (1 or 2) and the relative absence of activity for the other ipsilateral channel (4 or 3, respectively). **(C1)** Integrated “burstlet” activity (τ = 3 ms) and **(C2)** corresponding cross-correlation plot for same animal as “**B**”.

**Figure 10:**
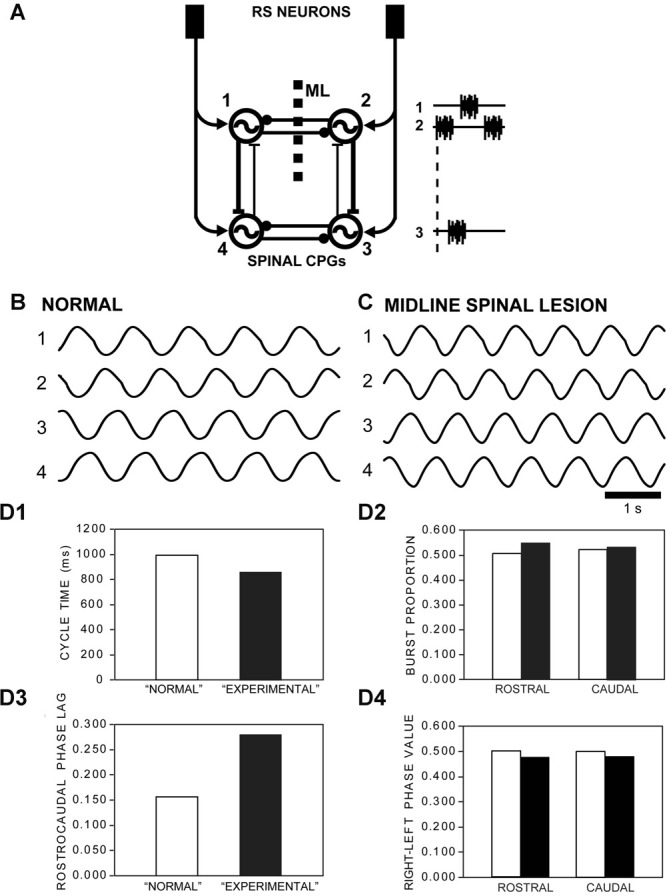
**(A)** Diagram of computer model showing RS neurons in the brain directly activating rostral (1, 2) and caudal (3, 4) left-right pairs of spinal oscillators that were connected by *net* reciprocal inhibition (–●). Ipsilateral oscillators were connected by asymmetrical reciprocal excitation (–|) that was stronger in the descending direction (*D*_E_ = 1.0) than the ascending direction (*A*_E_ = 0.24), similar to that previously described (Hagevik and McClellan, 1994; McClellan and Hagevik, 1999). The model representing “normal” animals (see **B**), without a longitudinal midline (ML) spinal lesion, had intact reciprocal inhibition between the rostral pair of oscillators. For the model representing “experimental” animals (see **C**), with a ML spinal lesion, reciprocal inhibition between the rostral pair of oscillators was removed.** (B)** Rhythmic “locomotor” output waveforms generated by a model representing “normal” animals were characterized by left-right alternation (1↔2, 3↔4) and a rostrocaudal phase lag (1→4, 2→3). **(C)** “Locomotor” output waveforms from a model representing “experimental” animals also featured left-right alternation and rostrocaudal phase lags, but with some differences in locomotor parameters. **(D)** Parameters of rhythmic “locomotor” output waveforms generated by the computer models representing “normal” animals (open bars) and “experimental” animals with a rostral ML spinal cord lesion (black bars): **(D1)** CTs decreased by a moderate amount (~15%) for the “experimental” model (i.e., with rostral ML spinal lesion) compared to those for the “normal” model. **(D2)** BPs for rostral and caudal “locomotor” waveforms were modestly larger (~9% and ~2%, respectively) for the “experimental” vs. “normal” models. **(D3)** The rostrocaudal phase lag was substantially larger (~80%) for the “experimental” model vs. “normal” model. **(D4)** Right-left phase values decreased very modestly (~4.5%) following incorporation of a ML spinal lesion for the “experimental” model.

Several lamprey studies have examined if left and right locomotor CPG modules are rhythmogenic (autonomous) or if the modules are interdependent (Figure 1). Previous results from larval lamprey studies suggest that isolated left and right spinal CPG modules are not autonomous and require connections with other modules in intact parts of the spinal cord to generate rhythmic locomotor burst activity (Jackson et al., 2005). For adult lampreys, different studies support each of the above two possibilities (Buchanan and McPherson, 1995; Buchanan, 1999; Cangiano and Grillner, 2003, 2005; also see Soffe, 1989; Moult et al., 2013; Li et al., 2014; for results from *Xenopus*).

In the present study, the contribution of reciprocal connections between left and right spinal CPG modules to locomotor burst activity was tested in experimental adult lampreys with two spinal cord lesion paradigms (see Figures 3A–5A, 8A, 9A): (a) longitudinal midline (ML) lesion alone in the rostral spinal cord; and (b) rostral ML spinal lesion as well as a spinal transection (T) at the caudal end of the ML lesion. Importantly, in the present study actual behavioral responses and muscle activity were initiated via descending brain-spinal cord pathways in response to sensory stimulation of the oral hood (i.e., anterior head). For experimental animals, muscle activity was recorded and compared to that during swimming for normal animals. The results suggest that for adult lampreys, as was the case for larval animals (Jackson et al., 2005), reciprocal coupling between left and right spinal locomotor CPG modules contributes to both motor pattern phase control of burst activity as well as rhythmogenesis. Parts of this study were presented in abstract form (McClellan et al., 2015).

## Materials and Methods

### Animal Care

Young adult sea lampreys (*Petromyzon marinus*) were used for all experiments and were maintained in ~10 liter aquaria at ~23°C. The procedures in this study have been approved by the Animal Use and Care Committee at the University of Missouri.

### Longitudinal Midline Spinal Cord Lesions and Spinal Cord Transections

Most of the experiments were performed for two groups of adult lampreys: (a) normal animals for which muscle activity was recorded (*n* = 15; 120–175 mm; see Figure 2A); and (b) experimental animals with a rostral longitudinal midline (ML) spinal lesion from 13→35% body length (BL, normalized distance from anterior tip of head) for which muscle activity was recorded before and after a caudal spinal cord transection (T) at 35% BL (*n* = 21; 132–175 mm; see Figures 3A–5A, 8A, 9A). The position and extent of the ML spinal lesion and position of the T spinal lesion were based on a previous similar study that tested the rhythmicity of spinal CPGs for larval lampreys (Jackson et al., 2007). For several additional experimental animals (*n* = 4; 150–165 mm), muscle activity only was recorded following a longitudinal ML spinal cord lesion. The animal lengths for experimental animals were not significantly different compared to those for normal animals (unpaired *t*-test; InStat, Inc., La Jolla, CA, USA). For experimental animals, the purpose of the ML spinal lesion was to interrupt reciprocal coupling entirely between rostral left and right spinal CPG networks and to test if rostral locomotor muscle burst activity could be generated in the absence of this coupling (see Figure 10A). The purpose of the subsequent spinal cord transection (T) was to block ascending inputs from intact caudal spinal cord from affecting rhythmogenesis of rostral hemi-spinal cords and test if these hemi-spinal cords, disconnected from intact cord, could generate locomotor activity. In addition, for experimental animals, recording locomotor muscle burst activity after the ML spinal lesion alone ensured that the rostral muscle recording electrodes were properly positioned following the subsequent spinal cord transection (T).

Experimental lampreys were anesthetized in ~200 mg/l tricaine methanesulfonate (MS222; Crescent Research Chemicals, Phoenix, AZ, USA) and pinned dorsal side up in a dissection dish. Gauze was placed over the non-surgical areas of the body, and ice chips were placed on the gauze as well as along the sides of the animal for surgical areas of the body. A dorsal longitudinal midline incision was made from ~11% to 37% BL to expose the rostral spinal cord. The incision was held open with several retraction hooks made from modified #5–0 suture needles (Ethicon Inc.; Somerville, NJ, USA). A ML spinal cord lesion was made from ~13→35% BL (see thick horizontal lines in Figures 3A–5A, 8A, 9A) using a fine scalpel blade (Beaver “mini-blade” 376500, Arista Surgical Supply, New York, NY, USA). It should be noted that for adult lampreys, the midline of the spinal cord usually is clearly indicated by a blood vessel and sometimes by the central canal. The completeness of the midline spinal cord lesion was verified visually or, if necessary, by gently displacing the hemi-spinal cords laterally. Subsequently, the incision was pinched closed and secured with several small drops of cyanoacrylate (Super Glue Gel, Loctite Co.; Rocky Hill, CT, USA) that were evenly spaced along the longitudinal incision. Since animals with only ML spinal cord lesions were able to generate coordinated locomotor muscle burst activity, it is unlikely that glue leaked into the incision and affected spinal circuitry. Following ML spinal cord lesions, animals were placed in a container with aquarium water that was bubbled with oxygen for up to 1 h. Muscle recordings were performed either immediately after recovery from anesthesia or ~24 h later, but no substantial differences in behavioral capabilities were observed for these animals.

Following recordings of muscle activity (see below), 21 of 25 of the experimental animals with longitudinal ML spinal cord lesions were then re-anesthetized, and the spinal cord was exposed and transected at 35% BL (T; Figures 3A, 5A, 9A) so that muscle activity could be recorded before and after the transection in the same animal. Following spinal cord transections, animals were placed in a container with aquarium water that was bubbled with oxygen for up to 1 h, and muscle recordings were resumed following recovery from anesthesia.

### Recording of Muscle Burst Activity

Prior to insertion of muscle recording wires, normal animals or experimental animals with rostral ML spinal cord lesions were placed in a swim chamber (24 × 44 cm) and videotaped using an S-VHS camera (Panasonic PVS 770; Yokohama, Japan; 30 frames/s, 8 ms shutter speed) that was mounted ~133 cm above the animals. Importantly, actual swimming behavior and other behavioral responses were initiated by descending brain-spinal cord pathways in response to electrical stimulation (1–10 mA, 2 ms pulses at 100 Hz for 50 ms) or mechanical stimulation (forceps) applied to the oral hood (anterior head).

Subsequently, animals were anesthetized, and pairs of fine copper wires (60 μm diameter), insulated except at the tips, were inserted bilaterally into body musculature at the following locations: (a) 25% BL (electrodes 1, 2) and 45% BL (electrodes 3, 4; *n* = 21; Figures 2A–4A); or (b) 25% BL and 30% BL (*n* = 4; Figures 8A, 9A). The EMG wires, which were bundled together and sutured to the dorsal surface of the animals at ~15% BL, were attached to a rotating swivel arm positioned over the swim chamber, allowing the animals to move freely within the chamber without appreciable loading from the recording wires. Muscle activity was recorded (Model 1700; A-M Systems, Inc., Carlsborg, WA, USA), amplified (initial gain of 1000X), filtered (0.1–5.0 kHz), and then stored on VHS tape (NeuroData DR886, Cygnus Technologies, Delaware Water Gap, PA, USA; 11 kHz sampling rate per channel). Simultaneously, animal movements were recorded with an S-VHS camera, and a custom video frame counter synchronized in time the video frames and the muscle activity, as previously described (Davis et al., 1993; McClellan et al., 2016). After completion of the muscle recordings, animals were re-anesthetized, body lengths were measured, and the numbers of segments between ipsilateral recording electrodes were counted (1→4, 2→3; Figures 2A–4A, 8A, 9A).

The recorded muscle activity was played back and acquired using a custom data acquisition/analysis system (DT-3016 acquisition board, Data Translations, Marlboro, MA, USA; within each 2 ms sampling interval, the minimum and maximum voltage values of muscle activity were acquired and displayed), as previously described (Jackson et al., 2007; Shaw et al., 2010). Normal animals (*n* = 15) and experimental animals with only a longitudinal ML spinal cord lesion (*n* = 21) were able to swim and generated locomotor muscle burst activity. The following parameters of this activity were measured or calculated, as previously described (Davis et al., 1993; McClellan and Hagevik, 1997; Boyd and McClellan, 2002; McClellan et al., 2016): cycle times (CT) were defined as the interval between the onsets of consecutive bursts; burst proportions (BPs; BP1–BP4) were equal to the burst duration (onset-to-offset) for a given recording channel (1–4; Figures 2A–4A) divided by CT; intersegmental rostrocaudal phase lags (Φ_2_→_3_ or Φ_1_→_4_) were calculated as the ratio of the delay between the midpoints of ipsilateral bursts and CT, divided by the number of intervening segments; and right-left phase values (Φ_2–1_ or Φ_3–4_) were equal to the phase of the midpoint of a right burst relative to the CT defined by the midpoints of bursts on the left side.

For either normal animals (*n* = 15) or experimental animals with ML spinal lesions (*n* = 21), right and left BPs for a given level of the body (e.g., BP1 and BP2) were not significantly different (unpaired *t*-test; InStat) and therefore were averaged to yield BP_ROSTRAL_ (25% BL) and BP_CAUDAL_ (45% BL; Figure 3C). Similarly, right and left intersegmental rostrocaudal phase lags were not significantly different for normal animals or experimental animals with ML lesions (unpaired *t*-test), and were averaged. A given locomotor parameter for normal animals was compared to that for experimental animals with a ML spinal cord lesion using an unpaired *t*-test with Welch correction, when necessary, or Kruskal-Wallis with Dunn’s multiple comparisons post-test (Figure 3C).

Animals with both longitudinal ML spinal cord lesions and caudal spinal transections (T; Figures 3A, 5A) were not able to swim and usually did not produce locomotor-like muscle activity. However, the isolated left and/or right rostral hemi-spinal cords sometimes generated relatively high frequency muscle “burstlet” activity (Figures 5, 7). Because the muscle “burstlet” activity probably is analogous to the previously described *in vitro* “fast rhythm” (Cangiano and Grillner, 2003, 2005), which is thought to represent swimming activity, further analysis was performed on “burstlet” activity. When there were at least three consecutive “burstlet” cycles, CT, frequencies (*f* = 1/CT) and BPs were measured or calculated (Figure 6), similar to the methods described above for determining the parameters of locomotor muscle burst activity.

### Auto-Correlation and Cross-Correlation Analysis

First, for experimental animals with ML and T spinal lesions (*n* = 17; Figures 3A, 5A), 700, 1000, or 2000 ms duration recordings of rostral “burstlet” activity (e.g., Figure 5) were imported into a spreadsheet (Excel; Microsoft, Redman, WA, USA) and mathematically rectified and integrated (τ = 3–10 ms; first-order “leaky” integrator). Using the CORREL function in Excel (calculates the Pearson Product-Moment Correlation Coefficient; −1.0 to +1.0), the auto-correlation values vs. time lag were calculated for left and/or right “burstlet” activity. These values were shifted by +1, and the sum was divided by 2, such that an auto-correlation value of +1.0 indicated a positive correlation (e.g., in-phase waveforms), while a low value close to 0.0 indicated a negative correlation (e.g., out-of-phase waveforms). The validity of the above approach was confirmed by applying the algorithm to a mathematically generated half-sine wave. For the auto-correlation plots (Figure 7), the coefficient of rhythmicity was defined by C_r_ = (a1 − a2)/(a1 + a2), where a1 and a2 were the amplitudes of the second peak and first trough, respectively, as previously described (Cangiano and Grillner, 2003; Li et al., 2010). The frequency of rhythmic “burstlet” activity was calculated as the inverse of the x-axis coordinate of point a1 of the auto-correlation plots (Figure 7). Auto-correlation plots had to have at least four initial consecutive peaks to be considered evidence for rhythmicity.

Second, for experimental animals with ML and T spinal lesions (*n* = 17), 700, 1000, or 2000 ms duration recordings of rostral muscle “burstlet” activity (1 and 2 in Figure 5) were imported into Excel, and mathematically rectified and integrated. Using the CORREL function, cross-correlation values vs. time lag were calculated for rostral left and right “burstlet” activity (1 and 2 in Figure 5). The cross-correlation values were shifted and scaled, as described above, and used to detect possible left-right phase coupling. The validity of this approach was confirmed by applying the algorithm to two mathematically-generated half-sine waves of the same frequency that were phase shifted by different amounts. Cross-correlation plots had to have at least three initial consecutive peaks to be considered evidence for phase coupling.

Third, for some experimental animals with only ML spinal lesions (*n* = 4; Figure 8A), 700, 1000, or 2000 ms duration recordings of muscle activity were imported into Excel, and mathematically rectified and integrated (Figure 8B1). Using the CORREL function, cross-correlation values vs. time lag were calculated for ipsilateral activity recorded within the part of the body containing the ML spinal lesion (1→4, 2→3; Figure 8B2). The cross-correlation values were shifted and scaled, as described above, and used to detect possible rostrocaudal phase lags for ipsilateral burst activity. Finally, following subsequent spinal transection at 35% BL for the same animals, the above approach was repeated to determine if ipsilateral “burstlet” activity was accompanied by a rostrocaudal phase lag (Figure 9).

### Computer Modeling

An iterative computer model of locomotor CPGs in the lamprey spinal cord (Hagevik and McClellan, 1994) was used to simulate locomotor output for normal animals and for experimental animals with rostral longitudinal ML spinal cord lesions (Figure 10A). Spinal CPGs consisted of rostral (1, 2) and caudal (3, 4) left-right pairs of phase oscillators (Figure 10A), each representing the merged CPG networks for ~20 segment regions of spinal cord, as previously described (Hagevik and McClellan, 1994; McClellan and Hagevik, 1999). Left and right oscillator pairs (1↔2 or 3↔4) were coupled by *net* reciprocal inhibition (strength = −0.3), as previously described (Hagevik and McClellan, 1994; McClellan and Hagevik, 1997, 1999). Ipsilateral oscillators (1↔4 and 2↔3) were coupled by asymmetrical reciprocal excitation that was stronger in the descending direction (D_E_ = 1.0) than the ascending direction (A_E_ = 0.24), similar to that previously described (Hagevik and McClellan, 1994; McClellan and Hagevik, 1999). For the present model, the value for A_E_ was decreased slightly compared to that for previous larval lamprey modeling studies (Hagevik and McClellan, 1994) to yield a rostrocaudal phase lag of ~0.160, which was used for the phase lag initial conditions. This resulted in a predicted intersegmental phase lag of ~0.008 (= 0.160/20 seg.), which was similar to the average value for locomotor muscle burst activity (Figure 3C3). At a given point in time, the sum of the synaptic inputs to each phase oscillator (excitatory: sum > 0; inhibitory: sum < 0) was applied to an excitatory (PRC_E_) or inhibitory (PRC_I_) phase response curve (PRC), respectively (see Figure1B in Hagevik and McClellan, 1994), to determine the degree to which the phase of a given oscillator would be advanced or delayed, as previously described (Hagevik and McClellan, 1994). The phase of oscillator “j” at the “i + 1” point in time was given as

(1) $$\theta_{\text{i}+1}=\theta_{\text{i}}+\text{Δt}/{\text{T}}_{\text{j}}+\text{Δ}\theta_{\text{PRC}}$$

where θ_i_ was the oscillator phase at the previous point in time, Δt was the iterative time step (= 1 ms), T_j_ was the intrinsic CT of the “j” oscillator, Δt/T_j_ was the intrinsic increment in phase with each time step, and Δθ_PRC_ was the phase shift resulting from synaptic inputs to the oscillator. The “locomotor” output waveform generated by oscillator “j” at the “i + 1” point in time was given by

(2) $${\text{V}}_{\text{j}}={\text{A}}_{\text{j}}*\sin(2\pi \theta_{\text{i}+1})$$

where A_j_ (= 1.0) was the peak amplitude of the oscillator output waveforms. Thus, the model calculated the voltage waveform for each oscillator at a given point in time based on the conditions at the previous point in time (Hagevik and McClellan, 1994; McClellan and Hagevik, 1999).

For the model representing “normal” animals (Figure 10A; without a ML spinal lesion), the intrinsic CT of each oscillator was 0.85 s, which resulted in an overall CT for the entire CPG network of ~1.0 s (see Figure 10B). To represent “experimental” animals with a longitudinal ML spinal cord lesion (ML; Figure 10A), the left-right coupling between the rostral pair of oscillators (1↔2) was removed (see Figure 10C). This resulted in a rostrocaudal phase lag of 0.280, which was used for the phase lag initial conditions. The parameters of the “locomotor” output waveforms from the model (Figure 10D) were defined and determined similar to the methods described above for analyzing locomotor muscle burst activity (Figure 3C). It should be noted that because the phase oscillators in the present model are rhythmogenic and can function in isolation, it was not possible to mimic the experimental conditions following both longitudinal ML spinal lesions and caudal spinal transections (e.g., Figures 3A, 5A).

## Results

### Normal Animals with Intact Coupling Between Left and Right Spinal Oscillators

For normal adult lampreys (*n* = 15 animals), swimming occurred spontaneously or could be elicited by stimulation of the oral hood (anterior head). Swimming was characterized by left-right bending at each level of the body and body undulations (S-waves) that propagated toward the tail with increasing amplitude, as previously described (Davis et al., 1993; McClellan et al., 2016). Swimming movements were produced by locomotor muscle burst activity (average of ~50 cycles per animal) consisting of left-right alternation of muscle activity for both rostral and caudal regions of the body (1↔2 and 3↔4) as well as a rostrocaudal phase lag for ipsilateral muscle activity (1→4 and 2→3; Figure 2) (Boyd and McClellan, 2002). Expanded regions of recorded locomotor muscle burst activity indicated that each burst usually consisted of a mostly continuous sequence of muscle action potentials without suggestions of regularly spaced high-frequency “burstlets”, such as those that occurred for experimental animals following ML spinal lesions or ML lesions and spinal transections (see below and Figures 4, 5).

### Experimental Animals with Rostral Longitudinal Midline Spinal Lesions

For experimental adult lampreys, rostral longitudinal ML spinal cord lesions (13→35% BL, horizontal line in Figure 3A) were performed to interrupt reciprocal coupling entirely between rostral left and right spinal CPG networks and to test if rostral locomotor muscle burst activity could be generated in the absence of this coupling (see diagram in Figure 10A). The position and extent of the ML spinal lesion was based on a previous similar study that tested the rhythmicity of spinal CPGs for larval lampreys (Jackson et al., 2007). Following ML spinal lesions, swimming-like movements could occur spontaneously or could be elicited by sensory stimulation of the oral hood and, in both cases, resulted in forward progression of the body. However, there often were several clear behavioral deficits: (a) lower than normal velocity of swimming; (b) difficulty with directional control of swimming; (c) lower than normal amplitude of left-right bending of the rostral part of the body; and (d) lower success rate for sensory-evoked episodes of swimming than for normal animals.

Experimental animals with rostral midline spinal lesions, but without spinal cord transections at 35% BL, generated two possible patterns of muscle burst activity. First, for one set of experimental animal (*n* = 21), stimulation of the oral hood elicited an average of ~34 cycles per animal of analyzable locomotor muscle burst activity, which consisted of left-right alternating burst activity for both rostral (1↔2) and caudal (3↔4) regions of the body, as well as a rostrocaudal phase lag for ipsilateral burst activity (2→3 and 1→4; Figure 3B1). However, each experimental animal often generated additional cycles of swimming in which the onsets and offsets of rostral muscle bursts were not well delineated, and therefore these cycles of locomotor activity were not analyzed. During episodes of locomotor movements, the amplitudes of rostral muscle burst activity were variable and often could be much larger (up to ~4 mV p-p at source) than those for the more caudal body. For all experimental animals with ML spinal lesions alone, at least some relatively high-frequency rostral “burstlet” activity was present within the longer rostral locomotor bursts (see Figure 8B1). In some cases, shortly after sensory stimulation, muscle burst activity sometimes initially was present only on one side of the rostral body and tonic on the contralateral side, and then subsequently transitioned into rostral left-right alternating activity.

During swimming, average CTs were shorter, but not significantly different (*p* = 0.15), for experimental animals with longitudinal ML spinal lesions compared to those for normal animals (Figure 3C1; unpaired *t*-test). For experimental animals, BPs for locomotor activity in the rostral and caudal body were significantly larger compared to those for normal animals (Figure 3C2). In addition, rostrocaudal phase lags were larger for experimental animals compared to those for normal animals (Figure 3C3). In contrast, for experimental animals right-left phase values for rostral and caudal locomotor burst activity were slightly decreased (~1%–2%) but not significantly different compared to those for normal animals (Figure 3C4).

Second, for an additional 10 experimental animals with only rostral ML spinal lesions, alternating muscle burst activity was mostly present just for caudal regions of the body in which the spinal cord was intact (Figure 4; data not included in Figure 3C). For the rostral body, where the midline spinal cord lesion interrupted left-right coupling between spinal CPG modules, left-right alternating locomotor burst activity was absent or very infrequently observed (data not shown). However, despite the absence of rostral locomotor activity, almost all of these animals displayed at least some relatively high-frequency rostral “burstlet” activity (Figure 4; see description below, and Figure 5).

### Experimental Animals with Rostral Midline Spinal Lesions and Spinal Transections

For most experimental animals with rostral ML spinal lesions (13%–35% BL) that generated locomotor muscle burst activity, the spinal cord was subsequently transected at 35% BL (T; Figures 3A, 5A; *n* = 17). The purpose of the subsequent spinal cord transection (T) was to block ascending inputs from intact caudal spinal cord from affecting rhythmogenesis of rostral hemi-spinal cords and to test if these hemi-spinal cords, disconnected from intact cord, could generate locomotor activity. However, following both types of spinal lesions, stimulation of the oral hood usually elicited tonic flexure responses above the transection, but rhythmic left-right bending of the rostral part of the body usually was not observed, very similar to the results for similar experiments conducted with larval lampreys (Jackson et al., 2005).

Following both midline spinal lesions and spinal cord transections, coordinated locomotor-like muscle burst activity did not occur. Instead, for animals with these two spinal lesions, sensory stimulation of the oral hood elicited two types of rostral muscle activity. First, for ~70% of the trials in which sensory stimulation elicited an extended response, the evoked muscle activity that occurred with latencies ≤5 s was tonic or of long duration and could occur on one or both sides of the body (Figure 3B2). In addition to these sensory-evoked responses, tonic or long duration muscle activity also could occur spontaneously. Because muscles in the rostral body could display some unpatterned activity under these conditions, it is unlikely that CPG interneurons were rhythmically active but subthreshold for activating and/or modulating motoneurons. In particular, for animals with only midline lesions of the rostral spinal cord (13%–35% BL), left-right alternating muscle burst activity could be present in the rostral body (Figure 3B1) but was abolished in the same animals following a spinal transection at 35% BL (Figure 3B2).

Second, for ~30% of the trials in which sensory stimulation elicited an extended response, evoked muscle activity with latencies ≤5 s consisted of rhythmic, relatively high-frequency “burstlet” activity in the rostral body for all experimental animals (*n* = 17), and this type of activity also could occur spontaneously (Figure 5B). Because this high-frequency “burstlet” activity probably is analogous to the previously described *in vitro* “fast rhythm” (Cangiano and Grillner, 2003, 2005), which is thought to represent swimming activity, further analysis was performed on “burstlet” activity. The rostral “burstlet” activity could occur on one side or both sides of the body, but in the latter case, the right and left “burstlet” activity usually had different frequencies and a varying phase relationship, as indicated by cross-correlation analysis (not shown; see “Materials and Methods” section). The amplitudes of the “burstlet” activity varied from ~80 μV up to ~4 mV p-p at the source. Although the minimum frequency of the “burstlet” activity was ~5–10 Hz, which is in the upper range of swimming activity for adult lampreys (McClellan et al., 2016), the average frequency of the “burstlet” activity was 24.2 ± 4.7 Hz, and maximum frequencies could be 50 Hz or higher (Figures 6A1–C1). The average CT for “burstlet” activity (57.5 ± 28.5 ms) was significantly shorter than those during swimming for normal animals or experimental animals with only ML spinal lesions (*p* < 0.001; Kruskal-Wallis with Dunn’s multiple comparisons post-test). BPs for “burstlet” activity had an average value of 0.607 ± 0.070, and were significantly larger than those during swimming for normal animals (*p* < 0.001; Kruskal Wallis with Dunn’s multiple comparisons post-test) or for experimental animals with only ML spinal lesions (*p* < 0.05; Kruskal-Wallis with Dunn’s multiple comparisons post-test). For “burstlet” activity, 19 of 33 plots (~58%) of BP vs. CT had statistically significant negative slopes (Figures 6A2–C2), while 4 of 33 plots (~12%) had significant positive slopes (linear regression analysis; InStat). However, 12 of the 23 plots with slopes that were significantly different from zero were better described by a curve than a line (Runs test; InStat).

The “burstlet” activity was not stereotypic, and CTs or frequencies often were variable from one cycle to the next. In addition, several consecutive “burstlets” often occurred together and then were interrupted by tonic activity, a longer-than-usual “burstlet”, or single action potentials. This variability of the frequency for many episodes of “burstlet” activity made auto-correlation analysis problematic. For certain episodes, the “burstlet” activity was quite regular and resulted in relatively unambiguous auto-correlation plots (Figures 7A,B). Overall, the average coefficient of rhythmicity for analyzable episodes of “burstlet” activity was C_r_ = 0.26 ± 0.13, and the average frequency for this particular activity was Freq = 25.2 ± 10.9 Hz (*n* = 17 animals, 189 episodes).

### Coordination of “Burstlet” Motor Activity within Hemi-Spinal Cords

Following ML spinal lesions and spinal transections (T), most of the “burstlet” activity had frequencies much higher than those typical of swimming for adult lampreys (Figures 6, 7). To further investigate the function of “burstlet” activity, rostral and caudal muscle recording electrodes were positioned within a region of the body encompassed by the ML spinal lesion (Figure 8A; *n* = 4). Following ML spinal lesions, but prior to caudal spinal transections, integrated locomotor muscle burst activity was characterized by left-right alternation (1↔2, 3↔4) and a rostrocaudal phase lag (1→4, 2→3; Figure 8B1). However, “burstlet” activity often was superimposed on the longer locomotor bursts (arrowheads in Figure 8B1). Cross-correlation plots of ipsilateral burst activity (Figure 8B2) yielded an average frequency of 4.13 ± 1.12 Hz (CT ≈ 240 ms) and an average rostrocaudal phase lag of 0.0107 ± 0.0122 (*n* = 4 animals, 74 episodes).

Subsequently, following a spinal cord transection (T) at 35% BL for the same animals (Figure 9A), rhythmic “burstlet” activity could be recorded from within the body region encompassing the spinal ML lesion (Figures 9B1,C1). For some episodes, “burstlet” activity was present for one ipsilateral channel (1 or 2) but was mostly absent for the other ipsilateral channel (4 or 3, respectively) (first half of Figure 9B1). In addition, most of the cross-correlation plots did not indicate a clear phase lag for ipsilateral “burstlet” activity (Figures 9B2,C2). For 8 of the 62 selected episodes that had several consecutive cycles (Freq = 26.41 ± 11.59 Hz), the cross-correlation plots provided some possible indications of ipsilateral phase coupling, but with a relatively high average rostrocaudal phase lag of 0.0248 ± 0.0416 (*n* = 4, 8/31 episodes). However, a visual inspection of these particular recordings often revealed that several-to-many of the “burstlets” for one ipsilateral channel were not time-locked with those for the other ipsilateral channel. Thus, the apparent phase coupling suggested by the cross-correlation plots might have been due to a few consecutive ipsilateral “burstlets” that were coupled infrequently or a few consecutive ipsilateral “burstlets” that had similar but not identical frequencies. Also, for one episode in particular with multiple, repetitive “burstlet” activity for one ipsilateral channel and a single “burstlet” for the other ipsilateral channel, the resultant cross-correlation plot was cyclic vs. time lag. Thus, cross-correlation plots alone can be misleading and must be interpreted carefully, including a visual inspection of the original recordings.

### Computer Models Representing Normal Animals and Experimental Animals

The computer model of spinal CPGs representing “normal” lampreys (i.e., without a ML spinal lesion; Figure 10A) generated a rhythmic “locomotor” output pattern that consisted of left-right alternation (1↔2 and 3↔4) and a rostrocaudal phase lag (1→4 and 2→3; Figure 10B). The overall CT of the “locomotor” output was ~1.0 s (Figures 10B,D1), indicating that the various connections between the CPG oscillators resulted in a slower rhythm than the intrinsic CT of 0.85 s for each oscillator, as previously shown (Hagevik and McClellan, 1994). In addition, the rostrocaudal phase lag was ~0.160, and because rostral and caudal parts of the model are assumed to represent ~20 spinal segments (see Hagevik and McClellan, 1994), the intersegmental phase lag was predicted to be ~0.0080 (= 0.160/20 seg.), which is similar to the average phase lag for locomotor muscle burst activity for normal animals (Figure 3C3).

To represent “experimental” animals with a rostral longitudinal ML spinal cord lesion (e.g., Figure 3A), the reciprocal connections between the rostral pair of oscillators (1, 2) in the model were removed (Figure 10A). Under these conditions, the overall CT of the “locomotor” output waveforms decreased by ~15% compared to that for the simulations representing “normal” animals (Figures 10B,C,D1), similar to the ~20% decrease in this parameter for locomotor muscle burst activity (Figure 3C1). The rostral and caudal BPs increased by ~9% and ~2%, respectively, following incorporation of the ML spinal lesion in the model (Figure 10D2), while there was a ~50% and ~15% increase, respectively, in this parameter for locomotor muscle burst activity (Figure 3C). Following implementation of the ML spinal lesion in the model, the rostrocaudal phase lag of the “locomotor” output waveforms increased by ~80% (Figure 10D3), in general similar to the ~35% increase in this parameter for the biological results (Figure 3C3). Finally, after incorporation of the rostral ML spinal lesion in the model representing experimental animals, the “locomotor” output waveforms displayed a relatively small decrease (~4.5%) for both rostral and caudal right-left phase values (Figure 10D4), while the muscle recording data indicated non-significant differences (~1%–2%) for this parameter between normal animals and experimental animals (Figure 3C4). In summary, implementation of a rostral ML spinal lesion in the computer model resulted in changes in the parameters of the “locomotor” output waveforms (Figure 10D) that were similar to many, but not all, of the differences for locomotor muscle burst activity observed between normal and experimental animals (Figure 3C). Unfortunately, because the phase oscillators in the present model are rhythmogenic and can function in isolation, it was not possible to mimic the experimental conditions following both longitudinal ML spinal lesions and caudal spinal transections (T).

## Discussion

### Role of Left-Right Reciprocal Connections for Adult Lamprey Spinal Locomotor Networks

First, in the present study with adult lampreys, a longitudinal midline (ML) spinal cord lesion (13%–35% BL) was used to interrupt left-right reciprocal coupling between left and right CPG modules in the rostral spinal cord. Importantly, in the present study actual locomotor movements and muscle burst activity, as well as other motor activity, were initiated in whole animals by descending brain-spinal pathways in response to sensory stimulation of the anterior head. For the majority of animals following this type of lesion, sensory stimulation of the oral hood (anterior head) elicited locomotor movements and locomotor muscle burst activity with left-right alternation for both the rostral and caudal body as well as a rostrocaudal phase lag (Figure 3). For these experimental animals, BPs and rostrocaudal phase lags for locomotor muscle burst activity were significantly larger than those for normal animals. Also, for experimental animals, average CTs were shorter, but not significantly different (*p* = 0.15), compared to those for normal animals.

A computer model representing spinal CPGs for normal animals and experimental animals with a rostral ML spinal lesion generated “locomotor” output waveforms (Figure 10) that mimicked several but not all of the differences in locomotor muscle burst activity from the biological experiments (Figure 3C). For example, removing left-right coupling between the rostral pair of oscillators will decrease the intrinsic CT for this oscillator pair, as previously demonstrated (see Hagevik and McClellan, 1994). Because of the dominant descending ipsilateral excitatory coupling in the model (Figure 10A), the above change will decrease overall CTs for the entire CPG network (Figure 10D1), although experimentally this change was not quite significant (*p* = 0.15; Figure 3C1). Also, a decrease in the intrinsic CT for the rostral oscillator pair relative to that for the caudal oscillator pair will increase rostrocaudal phase lags (Figure 10D3), as also shown experimentally (Figure 3C3). Finally, without reciprocal inhibition contributing to burst termination for the rostral pair of oscillators, BPs will increase for these oscillators and, to a lesser extent, for those of the caudal pair of oscillators (Figure 3C2). However, the model did not fully capture this aspect of the muscle activity patterns (Figure 10D2). Finally, following incorporation of the ML in the model, right-left phase values changed very little (Figure 10D4), as also shown experimentally (Figure 3C4). This basic phase oscillator model mimicked many of the neurobiological results for the present study, and has mimicked most of the neurobiological results for five previous studies: McClellan and Jang (1993); Hagevik and McClellan (1994); McClellan and Hagevik (1997, 1999); Benthall et al. (2017).

Second, a longitudinal ML spinal cord lesion (13%–35% BL) and a subsequent caudal spinal transection (T) at 35% BL were used to disconnect rostral hemi-spinal cords from intact caudal cord. For animals with these two spinal lesions, sensory stimulation of the oral hood usually elicited tonic, uncoordinated muscle activity in the rostral body (Figure 3B2) rather than locomotor-like muscle burst activity, very analogous to the results for similar experiments conducted with larval lampreys (Jackson et al., 2005). For the present study, it is unlikely that the absence of locomotor burst activity in isolated rostral hemi-spinal cords was due to excessive injury of spinal CPG modules. For example, for animals with only midline lesions of the rostral spinal cord (13%–35% BL), left-right alternating muscle burst activity could be present in the rostral body (Figure 3B1) but was abolished in the same animals following a spinal transection at 35% BL (Figure 3B2).

The evoked or spontaneous muscle “burstlet” activity in the present study very likely is analogous to the previously described electrically- or pharmacologically-induced *in vitro* “fast rhythm” (Cangiano and Grillner, 2003, 2005), which is thought to represent lamprey swimming activity. However, the muscle “burstlet” activity in the present study displayed several significant differences compared to locomotor muscle burst activity recorded from normal animals (Figure 2). First, the average frequency for “burstlet” activity was ~25 Hz, but individual frequencies could be >50 Hz (Figures 5, 6A1–C1, 7). These upper frequencies are much higher than those for locomotor muscle burst activity (McClellan et al., 2016). In addition, higher frequency and lower frequency “burstlet” activity appeared similar, except for differences in “burstlet” duration and number of action potentials per “burstlet”. Second, BPs for “burstlet” activity often varied significantly with CT (Figures 6A2–C2), instead of being relatively constant as during swimming (Wallén and Williams, 1984), and were significantly larger (mean ~0.600) than those for locomotor activity generated by normal animals or experimental animals with only a ML spinal lesion (Figure 3C2). Third, within the region of body containing the ML spinal lesion, “burstlet” activity could be present for one ipsilateral channel and absent for the other ipsilateral channel (first half of recordings in Figure 9B1). Fourth, there was very little convincing evidence for a locomotor-like rostrocaudal phase lag for ipsilateral “burstlet” activity (Figures 9B2,C2). Fifth, following ML spinal lesions alone, “burstlet” activity could occur in the presence (Figure 8B1) or absence (Figure 4) of locomotor activity, suggesting that the two were generated by different mechanisms. Sixth, for a subset of animals with ML spinal lesions alone that generated caudal but not rostral locomotor burst activity (*n* = 10; see “Results” section), substantial rostral “burstlet” activity often occurred (Figure 4). Therefore, the parameters and features of “burstlet” activity suggest that this activity does not correspond to locomotor muscle burst activity generated by spinal locomotor CPGs.

In summary, results from the present study suggest that for adult lampreys, as for larval lampreys (Jackson et al., 2005), left and right rostral hemi-spinal cords, disconnected from intact caudal spinal cord, are not able to reliably generate locomotor burst activity in response to sensory stimulation and descending activation from the brain. However, for animals with just a spinal transection at 35% BL, intact rostral CPG networks are fully capable of generating locomotor activity (Davis et al., 1993). Thus, for adult lampreys reciprocal connections between left and right spinal CPG modules appear to be necessary not only for left-right phasing of burst activity but also for rhythmogenesis itself.

### Other Locomotor Studies with the Lamprey or *Xenopus*

In previous studies with larval lampreys (Hagevik and McClellan, 1994) or adult lampreys (Cohen and Harris-Warrick, 1984; Alford and Williams, 1989), application of strychnine to the spinal cord converted left-right alternating locomotor burst activity to synchronous activity. These results suggest that left and right CPG modules are connected by relatively strong reciprocal inhibition in parallel with weaker reciprocal excitation, and the results from a computer model support this conclusion (Hagevik and McClellan, 1994). In addition, these results suggest that reciprocal inhibition mainly regulates left-right phasing of locomotor activity and is not critical for rhythmogenesis. However, in the presence of strychnine, left and right spinal CPG modules are still coupled by reciprocal excitation, and therefore these experiments do not specifically test if *isolated* left and right CPG modules can generate locomotor burst activity.

In one study using *in vitro* spinal cord from adult lampreys, a longitudinal midline lesion spanned about half the length of the spinal cord preparation, and pharmacological agents were applied to elicit motor activity. Under these conditions, left-right alternating ventral root burst activity was present in the intact part of the spinal cord but was largely abolished in the lesioned part of the cord (Buchanan, 1999). In separate studies, some of the CCI’s were photoablated in *in vitro* spinal cord preparations, and this manipulation altered the symmetry and CT of left-right bursting, and could eliminate bursting (Buchanan and McPherson, 1995). The above results were interpreted to mean that reciprocal coupling between left and right CPG modules, mediated in part by CCI’s, contributes to rhythmogenesis. In contrast, the results from other experiments using *in vitro* adult lamprey hemi-spinal cords, in which electrical or pharmacological stimulation was used to induce burst activity, suggest that hemi-spinal cords are rhythmogenic (Cangiano and Grillner, 2003, 2005).

For paralyzed preparations of embryonic *Xenopus*, left-right alternating motor activity, typical of swimming, as well as occasional synchronous activity were observed (Kahn and Roberts, 1982), suggesting that left and right CPG modules are rhythmogenic and left-right reciprocal inhibition largely controls phasing of motor activity. However, for both of the above motor patterns, left and right locomotor CPG modules still were coupled by left-right reciprocal connections and were not isolated. Following midline lesions of the *Xenopus* spinal cord, left or right spinal CPG modules were able to generate swimming-like motor activity in response to electrical stimulation of the rostral hemi-spinal cord, suggesting that these modules are rhythmogenic (Soffe, 1989). However, it is not known if *Xenopus* hemi-spinal cords can be induced to generate locomotor burst activity under more physiological methods for motor pattern initiation, such as in response to sensory stimulation and normal descending activation of spinal CPGs (however, see Li et al., 2010). More recently it has been shown that rapid silencing of activity on one side of the *Xenopus* spinal cord abolishes burst activity contralaterally, suggesting that reciprocal inhibition is important for generation of the normal locomotor rhythm (Moult et al., 2013).

### Comparable Studies with Other Animals

For crayfish, the right and left CPG modules in abdominal ganglia that control rhythmic swimmeret movements can function autonomously when isolated from other modules (Murchison et al., 1993). Likewise, for *Clione*, the right and left CPG modules that control alternating dorsal-ventral swimming movements of the “wings” can generate an alternating motor pattern when isolated from each other (reviewed in Arshavsky et al., 1998). Interestingly, the neural modules generating dorsal or ventral wing movements contain endogenous oscillator neurons. In contrast, for the leech, the interconnected left and right CPG circuits in segmental ganglia function as a unit for generating swimming motor activity (Friesen and Hocker, 2001), and certain ganglia do not generate swim activity when separated from the remaining ventral nerve cord (Hocker et al., 2000; also see Pearce and Friesen, 1985).

For mammalian quadrupedal locomotion studies, results from pharmacological or surgical manipulations suggest that distinct spinal “local control centers” or CPG modules control the rhythmic movements of each limb (reviewed in Orlovsky et al., 1999). First, for the isolated neonatal rat spinal cord, application of strychnine converts pharmacologically-induced left-right alternating locomotor-like burst activity to synchronous bursting (Cowley and Schmidt, 1995; also see Jovanović et al., 1999; for similar results for mudpuppy), suggesting that left and right spinal modules controlling each limb are rhythmogenic without the need for left-right reciprocal inhibition. Likewise, during development of embryonic rat, a switch in the sign of left-right reciprocal connections causes synchronous left-right spinal cord burst activity to transition to alternating activity (Nakayama et al., 2002). Furthermore, the CPG modules that control flexor and extensor rhythmic burst activity may also be rhythmogenic because application of strychnine to the isolated neonatal rat spinal cord converts flexor-extensor alternating activity to coactivation (Cowley and Schmidt, 1995). However, in the presence of strychnine, spinal CPG modules still appear to be coupled by reciprocal excitation, and therefore these experiments do not specifically test if *isolated* CPG modules can generate locomotor burst activity. Finally, for isolated neonatal mouse spinal cord, spontaneous or induced (electrical or pharmacological stimulation) rhythmic flexor or extensor bursts can occur without antagonistic motor activity (Whelan et al., 2000; also see Cheng et al., 1998; for complementary results in mudpuppy). However, the absence of ventral root bursting does not necessarily indicate a lack of interneuron activity in the corresponding CPG module (e.g., see Lafreniere-Roula and McCrea, 2005).

Second, surgically isolated right or left lumbar spinal cords from neonatal mice or rats can generate rhythmic locomotor-like burst activity in response to bath-applied pharmacological agents (Kudo and Yamada, 1987; Tao and Droge, 1992; Bracci et al., 1996; Cowley and Schmidt, 1997; Kjaerulff and Kiehn, 1997; Kremer and Lev-Tov, 1997; Bonnot and Morin, 1998; Whelan et al., 2000; Nakayama et al., 2002; reviewed in Bonnot et al., 2002; also see Cheng et al., 1998). However, in contrast to the above studies, results from activation of *in vitro* neonatal rat spinal motor networks via descending brain-spinal cord pathways, instead of pharmacological agents, suggest that commissural connections in the thoracolumbar spinal cord are critical for rhythmogenesis and generation of spinal locomotor-like burst activity (Cowley et al., 2009).

The *in vitro* spinal cord of the embryonic chick can generate spontaneous episodes of locomotor-like activity (O’Donovan, 1989). Following mid-sagittal lesions in the lumbosacral spinal cord, left or right spinal CPG modules are able to generate rhythmic burst activity (Ho and O’Donovan, 1993). Thus, for the embryonic chick spinal cord, left-right reciprocal connections do not appear to be necessary for rhythmogenesis.

For the spinal turtle, unilateral stimulation of different areas of the lower body elicits different specific variations of the rhythmic scratch reflex in the ipsilateral hindlimb (reviewed in Stein et al., 1998b), suggesting that separate left and right spinal CPG modules control scratching responses for each hindlimb. However, several additional results suggest that in response to unilateral stimulation, contralateral spinal circuitry contributes to ipsilateral scratch motor pattern generation (Stein et al., 1995, 1998a; Currie and Gonsalves, 1999; reviewed in Stein et al., 1998b). Finally, rhythmic hip flexor bursts can occur in the absence of extensor bursts, suggesting that reciprocal inhibition between flexor and extensor modules is not required for rhythmogenesis of hip flexor CPG modules (Stein et al., 1995, 1998a). This conclusion was further supported by extracellular recordings from interneurons associated with flexor and extensor motor activity (Stein et al., 2016).

### Overview of Other Studies

For the above studies, rhythmic motor activity was initiated or could occur as a result of several conditions: (a) pharmacological agents applied to the CPG networks; (b) motor activity occurred spontaneously; (c) activity evoked by electrical stimulation of CPG networks or possibly inputs to these networks; (d) activity evoked by sensory stimulation; or (e) motor activity evoked by brainstem/command stimulation. For almost all of the mammalian studies summarized above, pharmacological agents were applied to the isolated spinal cord to elicit spinal locomotor-like activity, and these studies support the autonomous CPG model hypothesis. Importantly, results from a mammalian study in which midline spinal lesions were performed and motor activity was initiated from the brain challenge the autonomous CPG model concept (Cowley et al., 2009). Thus, although artificial activation of CPG networks (e.g., pharmacological or electrical stimulation) is very convenient, these activation methods might not mimic all aspects of the normal initiation of rhythmic motor activity. Instead, these artificial activation methods might indicate what CPG networks are able to do under specific experimental conditions but not how the networks function under normal physiological conditions.

The spinal locomotor networks controlling a single limb likely are complex and might consist of multiple flexor-extensor half center networks, each of which controls flexor-extensor muscles acting around a different joint (hip, knee, ankle, etc.; Grillner, 1981). Thus, the rhythmicity of left and right CPG modules or flexor and extensor modules in the spinal cords of limbed vertebrates might not be directly comparable to that of the spinal CPGs in the lamprey, which are thought to consist of left and right modules that are reciprocally coupled.

## Summary

In the present study, longitudinal midline (ML) spinal cord lesions were made in the rostral spinal cords of adult lamprey to test the role of reciprocal coupling between left and right spinal CPG modules. Locomotor activity was initiated by descending brain-spinal cord pathways in response to sensory stimulation. Following a rostral ML spinal lesion, locomotor movements and locomotor muscle burst activity still could be initiated, but with some modifications of locomotor parameters, and a computer model mimicked several of these modifications. Following both a ML spinal lesion and caudal spinal transection (T), rostral left and right hemi-spinal cords, disconnected from intact caudal spinal cord, typically did not generate rhythmic locomotor-like burst activity in response to sensory stimulation and descending activation from the brain. In summary, for adult lampreys, reciprocal coupling between left and right spinal CPG modules appears to be important not only for left-right phasing of locomotor activity but also for rhythmogenesis. In addition, the present study indicates that extreme caution should be exercised when testing the operation of spinal locomotor networks using artificial activation of isolated or reduced nervous system preparations.

## Author Contributions

JAM, ASP, SH, WET and ADM: performed experiments, analyzed data, interpreted results, prepared figures and approved final version of manuscript. ADM: edited, revised manuscript and drafted manuscript.

## Conflict of Interest Statement

The authors declare that the research was conducted in the absence of any commercial or financial relationships that could be construed as a potential conflict of interest.
